# The use of pramipexole in drug-induced parkinsonism: A case study on a patient with bipolar depression

**DOI:** 10.1192/j.eurpsy.2022.1044

**Published:** 2022-09-01

**Authors:** L. Bueno Sanya, A. Bermejo Pastor, H. Andreu Gracia, O. De Juan Viladegut, L. Olivier Mayorga, I. Pacchiarotti

**Affiliations:** 1 Hospital Clinic de Barcelona, Department Of Psychiatry And Psychology, Institute Of Neuroscience, Barcelona, Spain; 2 Hospital Clinico San Carlos, Institute Of Psychiatry And Mental Health, Madrid, Spain

**Keywords:** pramipexole, bipolar depression, drug-induced parkinsonism, bipolar disorder type I

## Abstract

**Introduction:**

Pramipexole is a dopaminergic agonist used in the treatment of Parkinson’s disease and restless leg syndrome. Although there is a lack of pharmacological options to treat drug-induced parkinsonism, not many studies have been made on the use of pramipexole in its management. There is also evidence on pramipexole effectiveness on major depressive episodes, particularly for bipolar and treatment-resistant depression.

**Objectives:**

To describe a case of drug-induced parkinsonism treated with pramipexole in a complex patient with bipolar disorder type I and obsessive-compulsive disorder, long-term treated with antipsychotics and valproate.

**Methods:**

We present the case of a 51-year-old woman admitted in our psychiatric inpatient unit mainly to treat a bipolar depression. She also presented a parkinsonian syndrome, and a neurological study was conducted. As a negative DaTSCAN concluded its cause to be pharmacological, we decided to stop lurasidone and initiated pramipexole.

**Results:**

Guidelines suggest that drug-induced parkinsonism should be managed by discontinuing causative drugs or switching to another agent. However, we decided to use pramipexole with the aim of not only treating the parkinsonian syndrome but helping manage the depressive episode. We observed a remission of the depressive symptoms and an improvement in the parkinsonian symptoms.

**Conclusions:**

Although the best way to treat drug-induced parkinsonism is to avoid its causative agents, in clinical practice it is not always possible as some patients have resistant and complex psychiatric syndromes. We suggest considering pramipexole in its management, especially when dealing with a patient with a comorbid unipolar or bipolar depression. Further research is necessary to clarify its utility.

**Disclosure:**

No significant relationships.

